# Beyond the kernel: integrative phytochemical profiling and metabolic synergy of *Carya illinoinensis* byproducts in precision nutrition

**DOI:** 10.3389/fnut.2026.1843159

**Published:** 2026-07-17

**Authors:** Isabelle Soares, Robson Xavier Faria, Leandro Rocha

**Affiliations:** 1 Graduate Program in Applied Sciences for Health Products (PPG-CAPS), Faculty of Pharmacy of the Fluminense Federal University, Niterói, Brazil; 2 Laboratory for Environmental Health Assessment and Promotion (LAPSA), Oswaldo Cruz Institute, Rio de Janeiro, Brazil; 3 Laboratory of Natural Products Technology (LTPN), Department of Pharmaceutical Technology, Faculty of Pharmacy, Fluminense Federal University, Niterói, Brazil

**Keywords:** bioactive extraction, *Carya illinoinensis*, cultivar variability, functional nutrition, phytochemical fingerprinting, precision food science

## Abstract

The integration of agro-industrial byproducts into the human diet is pivotal for enhancing global food security and metabolic health. *Carya illinoinensis* (Pecan), traditionally valued for its edible kernels, possesses lignocellulosic byproducts (bark and shells) that are dense reservoirs of proanthocyanidins and phenolic acids. This review bridges the gap between agricultural genetic diversity and potential metabolic outcomes, proposing a circular economy model for the functional food industry. This study provides an integrated analysis of the phytochemical signature and nutritional characterization of pecan, emphasizing the impact of cultivar variability and advanced extraction technologies on bioactive yields. We decode the nutritional profile across different genotypes (e.g., “Barton,” “Pawnee,” and “Pecanita”), highlighting how genetic diversity influences (+)-catechin concentrations and the oleic/linoleic acid ratio. Furthermore, we examine the transition to “green extraction” technologies and their role in enhancing the stability and bioaccessibility of these metabolites. By matching phytochemical variability with targeted nutritional characteristics, this review establishes a scientific framework for the industrial valorization of pecans as a source of premium ingredients for functional nutrition.

## 1 Introduction

In the contemporary context of functional nutrition, the bioprospecting of plant-derived secondary metabolites to support human metabolic homeostasis has become a prominent scientific focus. *Carya illinoinensis* (Wangenh.) K. Koch transcending its classification as a high-calorie oilseed, functions as a complex biological system whose phytochemical profile interacts multifacetedly with physiological homeostasis. Although the lipid matrix of the edible endocarp (pecan kernel) is widely recognized—comprising between 70 and 79% of the dry weight—it is predominantly composed of oleic acid (55–75%). This composition provides significant nutritional benefits and confers on the pecan kernel oil oxidative stability comparable to or superior to that of extra virgin olive oil (1). The biological basis of this lipid synthesis has been elucidated through transcriptomic analyses, revealing key genes involved in fatty acid biosynthesis during embryonic development of this species (2).

Beyond the kernels, lignocellulosic byproducts, such as shells and bark, exhibit a phenolic density that is markedly superior to that of the edible portions. These tissues contain a vast array of bioactive compounds, including hydrolysable tannins, condensed tannins, flavanols, and ellagic acid derivatives. While current literature highlights the general health benefits of nut consumption (27), there remains a significant review gap regarding the systematic integration of cultivar-specific variation, advanced green extraction methodologies, and the exact grading of molecular evidence supporting the metabolic modulation of pecan byproducts. This lack of standardization hinders the industrial reproducibility and development of targeted functional ingredients.

To address this gap, this review provides a comprehensive synthesis of *Carya illinoinensis* byproducts. We detail the organ-specific phytochemical distribution, map the genotype-dependent metabolic plasticity across prominent cultivars, evaluate green extraction and stabilization frameworks, and critically grade the preclinical molecular evidence regarding gut-liver axis modulation. Finally, we discuss the sensory and safety bottlenecks preventing translational application in human nutrition.

## 2 Review methodology

To establish a rigorous, transparent synthesis of the literature, a narrative review methodology was executed. A systematic search of peer-reviewed articles published up to May 2026 was conducted using three primary electronic databases: PubMed, Scopus, and Web of Science. The search strategy employed combinations of the following keywords: (*Carya illinoinensis* OR pecan) AND (byproducts OR shell OR bark OR leaves) AND (phytochemicals OR polyphenols OR tannins) AND (microbiota OR gut-liver axis OR metabolic).

Inclusion criteria restricted selected articles to peer-reviewed original research and systematic reviews published in English, Portuguese, or Spanish, which focused specifically on the chemical characterization, extraction technologies, and biological activities of *C. illinoinensis* tissues. Studies evaluating non-specific plant mixtures, general forestry metrics, or those lacking clear botanical identification of the species were excluded. A total of 42 primary studies and key genomic/transcriptomic analyses were selected for detailed analysis and evidence grading.

## 3 Phytochemical profiling and nutritional composition

### 3.1 Compartmentation of bioactive metabolites

The distribution of bioactive compounds within *C. illinoinensis* is characterized by strict organ-specific compartmentalization (Supplementary Table S1). This spatial segregation likely evolved as an ecophysiological defense mechanism against pathogens and environmental stressors (3).

- *Pecan kernel (Endocarp)*: Focuses primarily on primary metabolism, storing a dense matrix of monounsaturated fatty acids (MUFAs) and polyunsaturated fatty acids (PUFAs), alongside lipophilic antioxidants such as tocopherols. Soluble phenolics, including gallic acid, (+)-catechin, and ellagic acid, are present but in lower concentrations compared to outer tissues (4).
- *Shell (Exocarp)*: This byproduct is a highly concentrated reservoir of condensed tannins (proanthocyanidins) and low-molecular-weight phenolic acids. Quantitative investigations demonstrate that *C. illinoinensis* shells possess total phenolic contents (TPC) ranging from 116 to 167 mg gallic acid equivalents per gram (mg GAE/g), and condensed tannin concentrations between 35 and 48 mg catechin equivalents per gram (mg CE/g). This phytochemical density yields robust antioxidant capacity, with free radical inhibition rates reaching 96% in ABTS, DPPH, and *β*-carotene/linoleic acid system assays (5, 6).
- *Testa (seed coat)*: Positioned between the shell and the kernel, the testa selectively accumulates high-molecular-weight ellagitannins and total phenolics, acting as a chemical barrier against oxidative degradation of the lipid-dense kernel (3).
- *Bark*: Comprises a complex lignocellulosic network rich in flavonols, polymeric tannins, and structural lignin. It represents a highly promising, yet underutilized, source of raw materials for compound encapsulations and controlled release due to its structural resilience (7).
- *Leaves*: In folk medicine, leaf infusions have been employed for antimicrobial applications (26). However, direct scientific literature on *C. illinoinensis* leaves remains limited compared to other parts of the tree. Preliminary studies indicate a high density of flavonols and derivates of the chalcone synthase pathway (8). To advance industrial feasibility, future research must systematically catalog the seasonal variation of leaf active principles, which currently remains a critical gap.

### 3.2 Macronutrient landscape and micronutrient synergy

From a nutritional perspective, the pecan kernel is defined by its highly dense lipid matrix, where oleic acid (C18:1) accounts for 55 to 75% of the total fatty acid composition, while linoleic acid (C18:2) represents the secondary PUFA fraction (36). In clinical and nutritional paradigms, replacing saturated dietary fats with this specific MUFA matrix is associated with reduced low-density lipoprotein (LDL) cholesterol and improved lipid profiles, directly mitigating the risk factors of atherosclerosis (9, 10, 35).

This lipid matrix does not operate in isolation; it benefits from local synergy with lipophilic micronutrients, notably *γ*-tocopherol is highly efficient at scavenging reactive nitrogen species and preventing the autoxidation of LDL cholesterol within cell membranes (11). Furthermore, the presence of essential minerals such as zinc (≈ 1.3 mg/28 g) and magnesium (≈ 37 mg/28 g) provides enzymatic cofactors that support endogenous antioxidant systems (such as superoxide dismutase, SOD). Phenolic compounds present in the kernel, such as gallic and ellagic acids, work in tandem with zinc to stabilize cell membranes against oxidative injury (12). The functional attributes of these primary tissues are summarized in Supplementary Table S2.

While these structural interactions suggest a preclinical bioactivity potential, these claims must not be equated to clinical efficacy. There are currently insufficient *in vivo* dose–response relationships or human clinical trials to establish a robust pharmacological profile for the direct management of chronic inflammatory states. Their consumption should be promoted as a supportive dietary strategy.

## 4 Cultivar and genotype-dependent variability

The phytochemical architecture of *Carya illinoinensis* is fundamentally governed by genetic determinants. Distinct genotypes exhibit unique secondary metabolite profiles, establishing a clear metabolic variability across the species (29) (Supplementary Table S3).

Comparative analyses of cultivars such as “Barton,” “Pawnee” “Pecanita” reveal how genetic lineage directs chemical synthesis:

- *“Barton”*: Characterized by elevated concentrations of catechins, condensed tannins, and anthocyanins, giving its byproducts exceptional free radical scavenging capacity (4).
- *“Pawnee”*: Features a superior oleic-to-linoleic acid ratio in its kernels, which confers heightened oxidative stability and longer shelf-life, alongside elevated levels of condensed tannins (13, 14).
- *“Pecanita”*: Exhibits high levels of gallic acid and a typical lipid profile well-suited for targeted phenolic extraction from its shells (2).

Beyond genetics, the “terroir” effect (soil chemistry, rainfall, and temperature fluctuations) modulates this phenotypic expression (30, 31). For example, cultivars grown under semi-arid conditions with elevated UV exposure show a compensatory upregulation of protective phenolic compounds in the exocarp (shells) compared to those cultivated in humid, subtropical climates (14).

At the molecular level, transcriptomic analyses have linked these phenotypic traits to specific genetic regulators. The expression of fatty acid desaturase genes (SAD and FAD) determines the degree of lipid unsaturation in the kernel (2), while the temporal expression on the chalcone synthase gene family (CiCHS) governs the accumulation of flavonoids during fruit maturation (8, 15). Consequently, selecting specific cultivars is a critical step in standardizing function food formulations.

## 5 Advanced extraction and stabilization technologies

### 5.1 Green extraction technologies vs. conventional methods

Standardizing bioactive extraction from the rigid, lignocellulosic matrix of pecan shells and bark requires transitioning from conventional thermal maceration to advanced green extraction technologies (32). Conventional methods require long exposure times and toxic organic solvents, which can degrade thermolabile monomers such as (+)-catechin. Advanced techniques minimize solvent consumption and process times while protecting bioactive structures (38) (Supplementary Table S4):

- *Ultrasound-assisted extraction (UAE)*: UAE uses acoustic cavitation, where the generation and collapse of microbubbles produce localized mechanical shear stress. This ruptures the tough plant cell walls, enhancing solvent penetration and mass transfer under mild temperatures (25–45 °C). UAE has proven highly effective for recovering sensitive monomeric phenolics from pecan shells without inducing thermal degradation (16).
- *Microwave-assisted extraction (MAE)*: MAE heats the matrix through dipolar rotation and ionic conduction. This rapid heating increases extraction kinetics but requires strict temperature control (typically below 60 °C) to prevent the degradation of heat-sensitive polyphenols (17, 33).
- *Supercritical fluid extraction (SC-CO_2_)*: Supercritical CO_2_ operates at low temperatures and near-ambient pressures, offering a highly selective, non-toxic pathway for extracting lipophilic compounds (e.g., tocopherols and kernel oil) (34). Adding polar modifiers (modifiers like ethanol) allows SC-CO_2_ to successfully recover moderately polar flavonoids while avoiding organic solvent residues (18, 19, 37).

Furthermore, tuning the solvent polarity (e.g., using ethanol-water binary mixtures) can alter the dielectric constant, allowing selective recovery of target compound classes (such as low-molecular-weight monomeric catechins versus larger condensed tannins) (20, 28).

### 5.2 Industrial stabilization: extrusion and microencapsulation

Once extracted, the highly reactive phenolic structures remain susceptible to oxidation, light, and gastrointestinal pH changes. Industrial stabilization technologies are critical to preserving their functional benefits:

- *Extrusion*: Applying high-temperature, short-time shear physical forces to pecan shells breaks down the rigid lignocellulosic polymers (cellulose and lignin). This increases the soluble dietary fiber fraction and soluble the bioaccessibility of bound phenolic acids, converting a tough agricultural residue into a highly functional dietary ingredient (6).
- *Microencapsulation*: Enclosing pecan polyphenols within biopolymeric matrices, such as zein (a hydrophobic maize protein), provides a protective physical barrier against degradation in the stomach’s acidic environment. This microencapsulation enables targeted, controlled release of action polyphenols in the intestinal lumen, enhancing their bioaccessibility and systemic persistence (7).

## 6 Molecular mechanisms of gut-liver axis modulation

The metabolic benefits of *C. illinoinensis* byproducts, particularly shells and bark, extend beyond simple antioxidant scavenging, involving complex orchestration of the gut-liver axis. In this section, we critically grade and evaluate the molecular mechanisms through which these bioactives exert their metabolic effects.

### 6.1 Prebiotic restructuring and gut barrier preservation

The low bioavailability of high-molecular-weight phytochemicals, such as condensed tannins (proanthocyanidins) and ellagitannins, means they transit the upper gastrointestinal tract relatively intact. Upon reaching the colon, they serve as selective substrates for microbial fermentation, acting as prebiotic agents.

- *Evidence grading (in vivo animal models)*: Preclinical studies in mice fed a high-fat diet have shown that pecan shell extracts stimulate a significant expansion of beneficial microbial taxa, particularly *Akkermansia muciniphila*, *Lactobacillus*, and *Bifidobacterium*, while suppressing opportunistic pathogens (21, 22).

*A. muciniphila* plays a key role in maintaining the integrity of the intestinal mucosal barrier by upregulating the expression of tight junction proteins, including Occludin and Zonula Occludens-1 (ZO-1). Strengthening these tight junctions prevents metabolic endotoxemia, which is characterized by the leakage of inflammatory lipopolysaccharides (LPS) from Gram-negative bacteria into the portal vein (23).

### 6.2 Attenuation of the gut-liver axis and metabolic secretions

Under obese or dysbiotic conditions, circulating LPS binds to Toll-like Receptor 4 (TLR4) on hepatic Kupffer cells, activating the nuclear factor kappa B (NF-κB) pathway and triggering the release of pro-inflammatory cytokines such as Tumor Necrosis Factor-alpha (TNF-*α*) and Interleukin-6 (IL-6).

- *Evidence grading (in vitro and in vivo animal models)*: Pecan byproduct extracts rich in ellagic acid and gallic acid have been shown to directly inhibit this inflammatory cascade. In cell models, these compounds downregulate TLR4 expression and prevent the nuclear translocation of NF-κB, mitigating downstream inflammatory signaling (24).

Additionally, the microbial fermentation of pecan fibers and non-extractable polyphenols yields short-chain fatty acids (SCFAs), notably butyrate and propionate. These SCFAs act as signaling ligands for G-Protein Coupled Receptors 41 and 43 (GPR41/43) on intestinal L-cells, triggering the secretion of glucagon-like peptide-1 (GLP-1) (25).

Circulating GLP-1 enhances pancreatic insulin secretion, delays gastric emptying, and improves peripheral insulin sensitivity. At the hepatic level, SCFA-mediated signaling activates Adenosine Monophosphate-Activated Protein Kinase (AMPK). This activation downregulates lipogenic genes such as Sterol Regulatory element-Binding Transcription Factor 1 (SREBP-1c), providing a molecular pathway for the reduction of hepatic steatosis (22).

These physiological interactions are represented structurally in Supplementary Figures S1, S2, tracking the pathway from initial intake to target organ modulation.

### 6.3 Grading the evidence: summary of experimental models

To avoid overstating these clinical outcomes, Supplementary Table S5 summarizes the current state of evidence for each mechanism, distinguishing between established preclinical findings and hypotheses requiring human validation.

## 7 Translational limitations and future perspectives

The nutritional and economic valorization of *Carya illinoinensis* byproducts represents a significant frontier in the circular bioeconomy. However, transitioning these agricultural residues from laboratory research to commercial functional ingredients requires addressing several critical bottlenecks:

1. *Absence of human clinical trials*: While the metabolic benefits of pecan shell and bark extracts–including gut-liver axis modulation and *Akkermansia* enrichment–are well-supported by *in vitro* and *in vivo* rodent models, double-blind, placebo-controlled human intervention trials are still needed. Clinical studies must validate safety, establish effective dosages, and confirm that these prebiotic benefits translate to humans.
2. *Sensory bitterness and astringency*: The high concentration of condensed tannins in pecan shells, while biologically beneficial, imparts a strong bitter and astringent taste. This can negatively impact consumer acceptance. Future research must optimize microencapsulation techniques (e.g., using zein, whey protein, or maltodextrin) to mask these flavors without reducing the bioaccessibility of the active compounds in the lower gastrointestinal tract.
3. *Food safety and contaminant screening*: Because shells and bark are external plant tissues, they are directly exposed to environmental contaminants. Industrial processing must establish rigorous screening protocols to ensure the final ingredients are free from heavy metals, pesticide residues, and mycotoxins (such as aflatoxins, which can accumulate during improper nut storage).
4. *Standardization of extract profiles*: Given that phytochemistry varies significantly between cultivars (such as “Barton” versus “Pawnee”) and across different growing regions, the food and supplement industries require standardized fingerprinted extracts to ensure consistent therapeutic potency.

By addressing these sensory, safety, and regulatory challenges, *Carya illinoinensis* byproducts can be successfully transformed from agricultural waste into high-value functional ingredients, bridging the gap between circular agriculture and human metabolic health.

## 8 Conclusion

The comprehensive evaluation of *Carya illinoinensis* highlights the species as a highly complex biological system whose functional value extends far beyond the lipid-dense edible kernel. While the kernel represents a premier source of cardioprotective monounsaturated fatty acids and synergistic tocopherols, this review demonstrated that the traditionally discarded lignocellulosic byproducts, specifically the shells and bark, serve as superior reservoirs of bioactive condensed tannins, proanthocyanidins, and phenolic acids. Preclinical evidence strongly supports the capacity of these polyphenolic extracts to modulate the gut-liver axis by acting as selective prebiotics that enrich beneficial microbial taxa, reinforce intestinal tight junctions, and mitigate systemic metabolic endotoxemia. At the cellular level, these interactions stimulate metabolic pathways involving AMPK activation and GLP-1 secretion, offering a plausible molecular basis for the protective effects of pecan byproducts against hepatic steatosis, chronic inflammation, and oxidative stress.

Despite these promising mechanisms, the therapeutic and industrial translation of *Carya illinoinensis* byproducts from laboratory-scale characterization to commercial functional ingredients remains subject to several critical limitations that must be addressed to ensure clinical and market viability. The clinical utility of these extracts is currently constrained by a pronounced lack of double-blind, placebo-controlled human intervention trials, leaving a significant gap between observed rodent phenotypes and verified human physiological outcomes. Furthermore, the high density of high-molecular-weight tannins responsible for the biological efficacy of the shells imparts severe astringency and bitterness, presenting a major sensory barrier that requires advanced stabilization techniques, such as biopolymer-based microencapsulation, to mask off-flavors without compromising intestinal bioaccessibility. These sensory and clinical challenges are further compounded by the necessity of stringent quality control protocols to screen external plant tissues for environmental contaminants, including heavy metals and storage-induced mycotoxins, alongside the mandatory requirement for high-resolution chromatographic standardization to reconcile the significant phytochemical variability across different cultivars.

Ultimately, bridging the gap between raw agricultural waste and standardized functional ingredients requires a multidisciplinary approach that integrates cultivar selection, green extraction technologies, and advanced food processing. Aligning the unique, genotype-dependent chemical fingerprints of cultivars like “Barton” and “Pawnee” with specific extraction and extrusion techniques will enable the development of highly stable and targeted nutraceuticals. By resolving these sensory, safety, and genetic standardization challenges in continuous research, the food and pharmaceutical industries can successfully transition *Carya illinoinensis* byproducts into valuable clinical assets, establishing a robust model for the circular bioeconomy that simultaneously advances sustainable agriculture and human metabolic health.
