# Supplementary material for: Beyond the kernel: integrative phytochemical profiling and metabolic synergy of Carya illinoinensis byproducts in precision nutrition
Source: Front Nutr. 2026 Jul 17;13:1843159. doi: 10.3389/fnut.2026.1843159 (PMC13423692; doi:10.3389/fnut.2026.1843159)
Supplement: Supplementary file 1 [file Supplementary_file_1.DOCX]

***Supplementary Material***

## Supplementary Tables and Figures

| \| Table 1: Organ-specific distribution of potential benefits, phytochemical rationale, and variability in *Carya illinoinensis*. \| \| \| \|  \| \| --- \| --- \| --- \| --- \| --- \| \| Plant Part \| Primary Bioactive Compounds \| Phytochemical Rationale \| Observed Variability \| References \| \| Kernel (Nut) \| MUFAs/PUFAs, tocopherols, flavonols, ellagic acid \| High concentration of unsaturated lipids and lipophilic antioxidants \| Strongly dependent on cultivar and post-harvest storage \| [RAJARAM *et al*., 2001; DOMINGUEZ-AVILA *et al.,* 2015] \| \| Shell (Byproduct) \| Condensed tannins (phoanthocyanidins) gallic acid \| Dense phenolic network, high antioxidant/radical scavenging capacity \| Varies significantly based on extraction methods and genetics \| [VILLASANTE *et al*., 2019; XU *et al*., 2020] \| \| Testa \| Tannins, high total phenolics \| Selective barrier of secondary metabolites \| Influences by seed maturation stage and environmental humidity \| [XU *et al*., 2020] \| \| Bark \| Flavonols, complex polymer phenolics \| Structural lignocellulosic matrix \| Influenced by tree age, seasonal peeling, and geography \| [KURECK *et al*., 2018 ; XU *et al.,* 2020] \| \| Leaf \| Flavonols, chalcone derivatives \| High density of solar-protective flavonoids \| Highly variable based on phenological stage and pest exposure \| [ZHANG *et al*., 2018; XU *et al*., 2020] \| \|  \|  \|  \|  \|  \|   Table 2: Phytochemical profile and functional attributes of *Carya illinoinensis* tissues. | | | |
| --- | --- | --- | --- | --- | --- | --- | --- | --- | --- | --- | --- | --- | --- | --- | --- | --- | --- | --- | --- | --- | --- | --- | --- | --- | --- | --- | --- | --- | --- | --- | --- | --- | --- | --- | --- | --- | --- | --- | --- | --- | --- | --- | --- |
| Plant Part | Primary Bioactive Compounds | Biological Properties & Functions | References |
| Kernel (Endorcap) | Lipids (MUFAs/PUFAs), Flavonols, Ellagic acid, catechins, Tocopherols | Antioxidant activity, metabolic modulation, and cardioprotection | [JIA *et al*., 2018 ; ZHANG *et al*., 2018] |
| Shell (Exocarp) | Condensed tannins, Gallic acid, Lignocellulose | High antioxidant capacity (5-20x higher than kernel), oxidative stress mitigation | [PINHEIRO DO PRADO *et al*., 2009 ; XU *et al.*, 2020] |
| Testa (Seed Coast) | Tannins, Total Phenolics | High density of secondary metabolites with anti-inflammatory potential | [XU *et al*., 2020] |
| Bark | Flavonols, Complex phenolic compounds | Structural source of metabolites for encapsulation and controlled release | [KURECK *et al*., 2018 ; XU *et al*., 2020] |

| Table 3: Therapeutic Recommendation Matrix for *Carya illinoinensis* Cultivars Based on Metabolic Targets. | | | |
| --- | --- | --- | --- |
| Target Condition | Recommended Cultivar | Primary Mechanism | Key Molecule |
| Atherosclerosis | ‘Pawnee’ | LDL Membrane Stabilization | Oleic Acid |
| Type 2 Diabetes | ‘Barton’ | α-glucosidase Inhibition | Catechins / Tannins |
| Oxidative Stress | ‘Barton’ / ‘Pecanita’ | Nrf2 Pathway Activation | Gallic Acid |

| Table 4: Synthesis of Technological Impact on the Bioactivity of *Carya illinoinensis*. | | | |
| --- | --- | --- | --- |
| Technology | Effect on the Matrix | Biological Consequence | Most Benefited Cultivar |
| Extrusion | Fiber depolymerization | Increases tannin bioaccessibility | ‘Barton’ |
| Encapsulation | Physical barrier (pH protection) | Increases serum stability of catechins | ‘Barton’ / ‘Pecanita’ |
| UAE Extraction | Acoustic cavitation | Increases MUFA and Vitamin E yield | ‘Pawnee’ |

Figure 1: Metabolic Flow: Pecan Polyphenols to GLP-1 Secretion. Detailed pathway showing: **1)** Pecan polyphenols (ellagitannins, flavonoids) entering the gut. **2)** Microbial fermentation increasing beneficial taxa (*A. muciniphila, Lactobacillus*). **3)** Fermentation yielding Short-Chain Fatty Acids (SCFAs) like Butyrate. **4)** SCFAs activating GPR41 / GPR43 receptors on L-cells. **5)** Upregulation of intracellular cAMP and Ca2+, stimulating GLP-1 secretion. **6)** Systemic effects: insulin production, satiety, and slowed gastric empying. Created by the author on the Canva website.


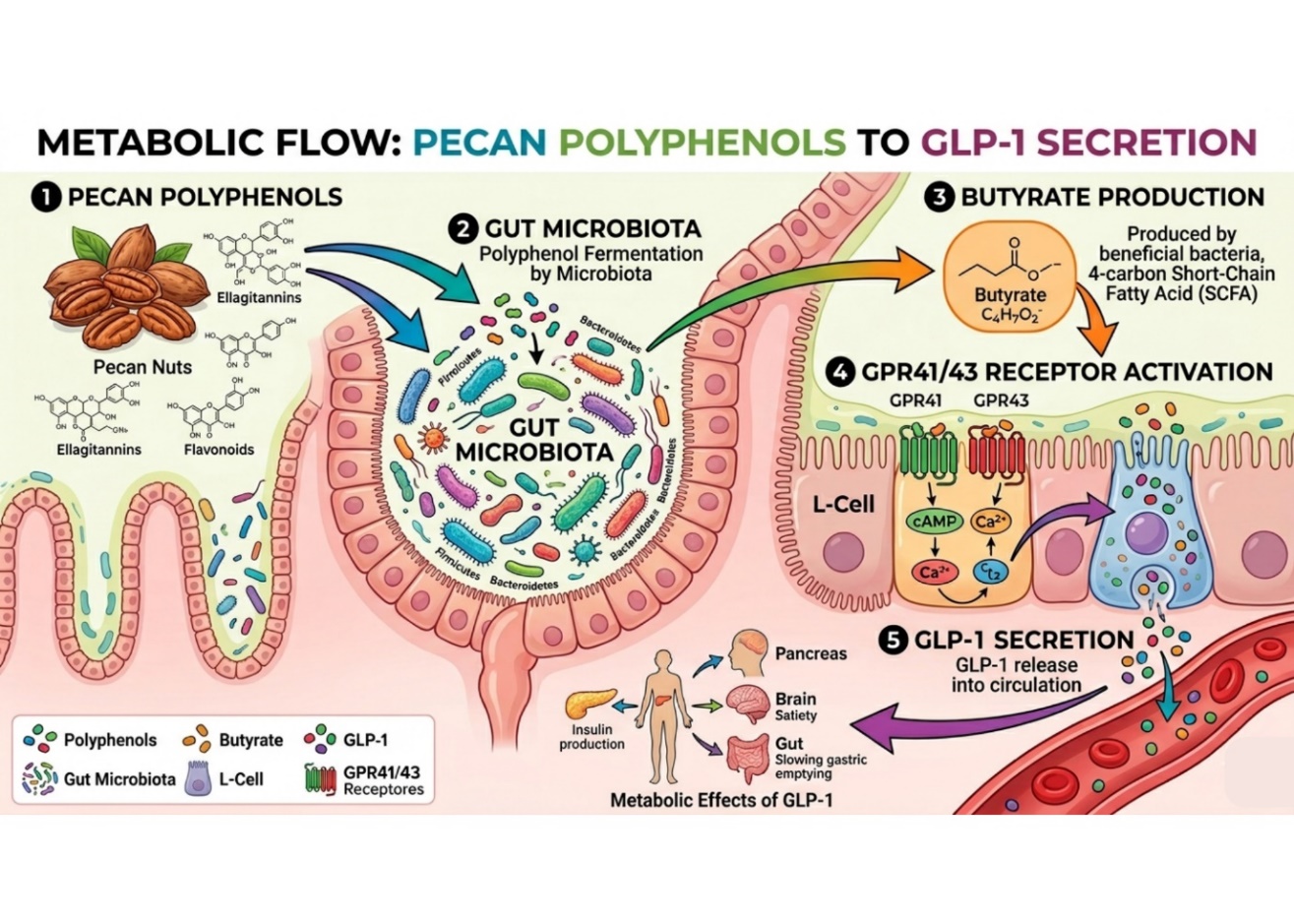


Figure 2: Integrative flowchart of therapeutic mechanisms. Consolidated pathway showing: **1)** Phytochemical matrix of *Carya illinoinensis* (Pecan). **2)** Three major therapeutic branches: - Oxidative Stress (Free radical neutralization, SOD/CAT upregulation; - Carbohydrate/Lipid Metabolism (Inhibition of alpha-glucosidase/lipase, PPARgamma/alpha activation); - Inflammatory Processes (NF-κB pathway blockage, decrease in TNF-alpha, IL-6). **3)** Concluding Clinical Outcomes (Cardiovascular protection, metabolic syndrome control, prevention of chronic diseases). Created by the author on the Canva website.


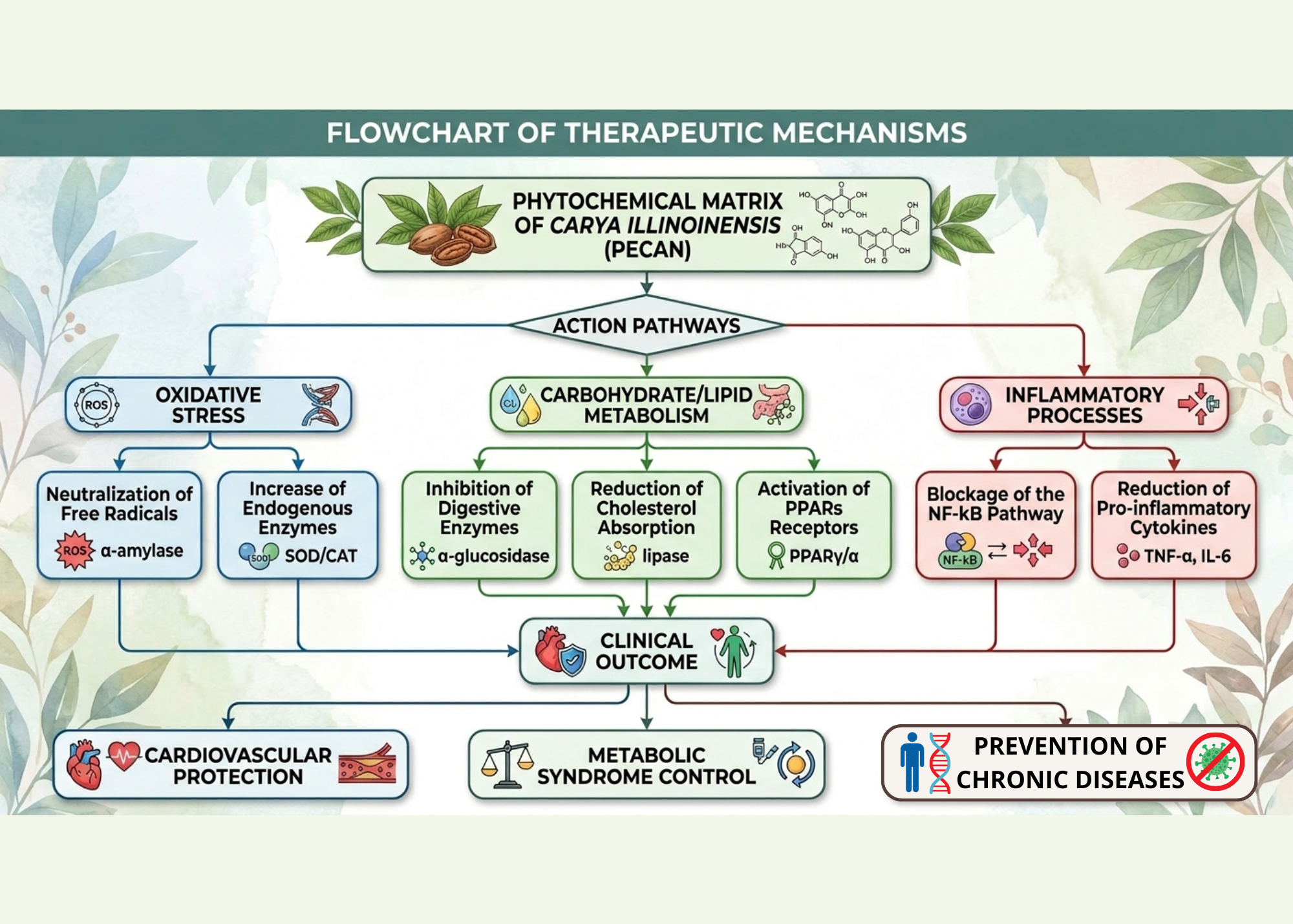


| Table 5: Summary of biological activities, molecular targets, and evidence grading for *Carya illinoinensis* bioactive compounds. | | | | |
| --- | --- | --- | --- | --- |
| Biological Activity | Key Bioactive Compounds | Primary Molecular Targets | Current Level of Evidence | References |
| Antioxidant | Gallic acid, catechins, proanthocyanidins | DDPH, ABTS, ROS scavenging; SOD, CAT enzymes | Strong Preclinical (Extensive *in vitro* & *in vivo* animal validation) | [VILLASANT *et al*., 2019; XU *et al*., 2020] |
| Anti-inflammatory | Flavonoids, phenolic acids | Nf-κB inhibition, COX-2, TNF-α, IL-6 | Moderate Preclinical (Validated in rodent obesity models and macrophage cell lines) | [ZHANG *et al*., 2018; XU *et al*., 2020] |
| Antidiabetic | Condensed tannins, soluble fibers | α-glucosidase and α-amylase enzymes | Moderate Preclinical (*In vitro* enzymatic inhibition; lacks human trials) | [YIN *et al*., 2020; XU *et al*., 2020] |
| Cardioprotective | Unsaturated fatty acids, tocopherols | LDL-c reduction, Apo-A1 and Apo-B modulation | Clinical Evidence (Established through human pecan kernel diets) | [RAJARAM *et al*., 2001; DOMÍNGUES-AVILA *et al*., 2015] |
| Antimicrobial | Tannins, leaf phenolics | Bacterial membrane disruption (Gram-positive) | In Vitro (Validated against isolated pathogens; no animal/human confirmation) | [XU *et al*., 2020; VILLASANTE *et al*., 2019] |
